# Bayesian Non-Parametric Inference for Multivariate Peaks-over-Threshold Models

**DOI:** 10.3390/e26040335

**Published:** 2024-04-14

**Authors:** Peter Trubey, Bruno Sansó

**Affiliations:** Department of Statistics, University of California, Santa Cruz, CA 95064, USA; bsanso@ucsc.edu

**Keywords:** multivariate extremes, peak over threshold models, bayesian non-parametric models, dirichlet process mixtures

## Abstract

We consider a constructive definition of the multivariate Pareto that factorizes the random vector into a radial component and an independent angular component. The former follows a univariate Pareto distribution, and the latter is defined on the surface of the positive orthant of the infinity norm unit hypercube. We propose a method for inferring the distribution of the angular component by identifying its support as the limit of the positive orthant of the unit *p*-norm spheres and introduce a projected gamma family of distributions defined through the normalization of a vector of independent random gammas to the space. This serves to construct a flexible family of distributions obtained as a Dirichlet process mixture of projected gammas. For model assessment, we discuss scoring methods appropriate to distributions on the unit hypercube. In particular, working with the energy score criterion, we develop a kernel metric that produces a proper scoring rule and presents a simulation study to compare different modeling choices using the proposed metric. Using our approach, we describe the dependence structure of extreme values in the integrated vapor transport (IVT), data describing the flow of atmospheric moisture along the coast of California. We find clear but heterogeneous geographical dependence.

## 1. Introduction

The statistical analysis of extreme values focuses on inferences for rare events that correspond to the tails of probability distributions. As such, it is a key ingredient in the risk assessment of phenomena that can have strong societal impacts like floods, heat waves, high concentration of pollutants, crashes in the financial markets, among others. The fundamental challenge of extreme value theory (EVT) is to use information, collected over limited periods of time, to extrapolate to long time horizons. This sets EVT apart from most of statistical inference, where the focus is on the bulk of the distribution. Extrapolation to the tails of the distributions is possible thanks to theoretical results that give asymptotic descriptions of the probability distributions of extreme values.

Inferential methods for the extreme values of univariate observations are well established, and software is widely available; see, for example, [1]. For variables in one dimension, the application of EVT methods considers the asymptotic distribution of either the maxima calculated for regular blocks of data, or the values that exceed a certain threshold. The former leads to a generalized extreme value (GEV) distribution that depends on three parameters. The latter leads to a generalized Pareto (GP) distribution, which depends on a shape and a scale parameter. Likelihood-based approaches to inference can be readily implemented in both cases. In the multivariate case, the GEV theory is well developed; see, for example, [2], but the inferential problem is complicated by the fact that there is no parametric representation of the GEV. This problem is inherited by the peaks over threshold (PoT) approach and compounded by the fact that there is no unique definition of an exceedance of a multivariate threshold, as there is an obvious dependence on the norm that is used to measure the size of a vector.

During the last decade or so, much work has been done in the exploration of the definition and properties of an appropriate generalization of the univariate GP distribution to a multivariate setting. To mention some of the papers on the topic, the work of [3] defines the generalized Pareto distribution, with further analysis of these classes of distributions presented in [4,5]. A recent review of the state of the art in multivariate peaks over threshold modeling using generalized Pareto is provided in [6] while [7] provides insight on the theoretical properties of possible parametrizations. These are used in [8] for likelihood-based models for PoT estimation. A frequently used method for describing dependence in multivariate distributions is to use a copula. Refs. [9,10] provide successful examples of this approach in an EVT framework.

Ref. [11] presents a constructive definition of the Pareto process, which generalizes the GP to an infinite-dimensional setting. It consists of decomposing the process into independent radial and angular components. Such an approach can be used in the finite-dimensional case, where the angular component contains information pertaining to the dependence structure of the random vector. Based on this definition, we present a novel approach for modeling the angular component with families of distributions that provide flexibility and can be applied in a moderately large dimensional setting. Our focus on the angular measure is similar to that in [12,13,14], which consider Bayesian non-parametric approaches. Yet, our approach differs in that it is established in the peaks-over-threshold regime and uses a constructive definition of the multivariate GP based on the infinity norm. The approach proposed in this paper adds to the growing literature on Bayesian models for multivariate extreme value analysis (see, for example, [12,13,14,15]), providing a model that has strong computational advantages due its structural simplicitly, achieves flexibility using a mixture model, and scales well to moderately large dimensions.

The remainder of this paper is outlined as follows. Section 2 comprises a brief review of multivariate PoT, detailing the separation of the radial measure from the angular measure. Section 3 details our approach for estimating the angular measure, based on transforming an arbitrary distribution supported in $R_{+}^{d}$ onto unit hyper-spheres defined by *p*-norms. Section 4 develops criteria for model selection in the support of the angular measure. Section 5 explores the efficacy of the proposed approach on a set of simulated data, and, acknowledging the relevance of extreme value theory to climatological events [16,17,18], estimates the extremal dependence structure for a measure of water vapor flow in the atmosphere, used for identifying atmospheric rivers. Finally, Section 6 presents our conclusions and discussion.

Throughout the paper, we adopt the operators ∧ to denote minima and the ∨ to denote maxima. Thus $\wedge_{i}s_{i}=\min_{i}s_{i}$, and $\vee_{i}s_{i}=\max_{i}s_{i}$. These operators can also be applied component-wise between vectors such as $a\wedge b=(a_{1}\wedge b_{1},a_{2}\wedge b_{2},\ldots )$. Similarly, we apply inequality and arithmetic operators operators to vectors. For example, a≤b, and interpret them component-wise. We use uppercase to indicate random variables, lowercase to indicate points, and bold-face to indicate vectors or matrices thereof.

## 2. A Multivariate PoT Model

To develop a multivariate PoT model for extreme values, consider a *d*-dimensional random vector $W=(W_{1},\ldots ,W_{d})$ with cumulative distribution *F*. A common assumption on W is that it is in the so-called domain of attraction of a multivariate max-stable distribution, *G*. Thus, following [7], assume that there exists sequences of vectors $a_{n}$ and $b_{n}$, such that $\lim_{n\to \infty }F^{n}\left(a_{n}w+b_{n}\right)=G\left(w\right)$. *G* is a *d*-variate generalized extreme value distribution. Notice that, even though the univariate marginals are obtained from a three-parameter family, there is no parametric form to represent *G*. Taking logarithms and expanding, we have that

$$\lim_{n\to \infty }n\left(1-F\left(a_{n}w+b_{n}\right)\right)=-\log G\left(w\right),$$

$\forall w\in R^{d}$ such that G(w)>0. It follows that

limn→∞Pran−1(W−bn)≤w∣W≤bn)=logG(w∧0)−logG(w)logG(0)=H(w),

where $a_{n}^{-1}$ indicates element-wise inversion, and {W≤bn} denotes the set where at least one coordinate is above the corresponding component of $b_{n}$. *H* is a multivariate Pareto distribution. It corresponds to a joint distribution conditional on exceeding a multivariate threshold. *H* is defined by a non-parametric function governing the multivariate dependence and two *d*-dimensional vectors of parameters that control the shapes and scales of the marginals. We denote these as ξ for the shapes and σ for the scales. Ref. [7] provides a number of stochastic representations for *H*. In this paper, we focus on a particular one that is proposed in [11]. To this end, we denote as *Z* a random variable with distribution *H* where ξ=1 and σ=0. Then, Z=RV where *R* and V are independent. $R={∥Z∥}_{\infty }=\vee_{i=1}^{d}Z_{i}$ is distributed as a standard Pareto random variable, and $V=Z/{∥Z∥}_{\infty }$ is a random vector in $S_{\infty }^{d-1}$, the positive orthant of the unit sphere under $L_{\infty }$ norm, with distribution Φ. This representation is central to the methods proposed in this paper. *R* and V are referred to, respectively, as the *radial* and *angular* components of *H*. The angular measure controls the dependence structure of Z in the tails. In view of this, to obtain a PoT model, we seek a flexible model for the distribution of $V\in S_{\infty }^{d-1}$, based on a Bayesian non-parametric model.

The approach considered in [6] focuses on the limiting conditional distribution *H*. An alternative approach to obtaining a limiting PoT distribution consists of assuming that regular variation (see, for example, [19]) holds for the limiting distribution of W, implying that

$$\lim_{n\to \infty }n\mathrm{Pr}\left[n^{-1}W\in A\right]=\mu \left(A\right),$$

for some measure μ that is referred to as the exponent measure. μ has the homogeneity property $\mu \left(tA\right)=t^{-1}\mu \left(A\right)$. Letting $\rho ={∥W∥}_{p},p>0$ and θ=W/ρ, define Ψ(B)=μ({w:ρ>1,θ∈B}), which is referred to as the angular measure. After some manipulations, we obtain that

(1) $$\lim_{r\to \infty }\mathrm{Pr}\left[\theta \in A|\rho >r\right]=\frac{\Psi (A)}{\Psi (S_{p}^{d-1})}.$$

Thus, a model for the exponent measure induces a model for the limiting distribution conditional on the observations being above a threshold defined with respect to their *p*-norm. The constraint that all marginals of μ correspond to a standard Pareto distribution leads to the so-called moment constraints on Ψ, consisting of

$$\int_{S_{p}^{d-1}}w_{i}\;d\Psi \left(w\right)=\frac{1}{d},\;\;i=1,\ldots ,d.$$

Inference for the limiting distribution of the exceedances needs to account for the normalizing constant in Equation (1) as well as the moment constraints. Because of these issues, in this paper, we prefer to follow the limiting conditional distribution approach. An example of the application of the regular variation approach using p=1 is developed in [13].

## 3. Estimation of the Angular Measure

To infer the PoT distribution, we consider two steps: First we estimate the shape and scale parameters for the multivariate Pareto distribution, using the univariate marginals; then we focus on the dependence structure in extreme regions by proposing a flexible model for the distribution of V. Consider $w_{i},\;i=1,\ldots ,n$ a collection of realizations of W. We start by setting a large threshold $b_{t,\ell }$ for the *ℓ*-th marginal, ℓ=1,…,d. Then, the distribution of $W_{\ell }$, conditional on exceeding the threshold, can be approximated with a generalized univariate Pareto. Thus,

$$\mathrm{Pr}\left[W_{\ell }>w_{i\ell }|W_{\ell }>b_{t,\ell }\right]={\left(1+\xi_{\ell }\frac{w_{i\ell }-b_{t,\ell }}{\sigma_{\ell }}\right)}_{+}^{-1/\xi_{\ell }}$$

where ${\left(\cdot \right)}_{+}$ indicates the positive part function. We set $b_{t,l}={\hat{F}}_{\ell }^{-1}\left(1-1/t\right)$, the empirical (1−1/t)-quantile. We then estimate $\xi_{\ell }$ and $\sigma_{\ell }$, for each *ℓ*, using likelihood-based methods. To estimate the angular distribution, we standardize each of the marginals. The standardization yields

(2) $$z_{i\ell }={\left(1+\xi_{\ell }\frac{w_{i\ell }-b_{t,\ell }}{\sigma_{\ell }}\right)}_{+}^{1/\xi_{\ell }}\;.$$

Note that $z_{i\ell }>1$ implies that $w_{i\ell }>b_{t,\ell }$, meaning that the observation $w_{i}$ is extreme in the *ℓ*-th dimension. Thus, $r_{i}={∥z_{i}∥}_{\infty }>1$ implies that at least one dimension has an extreme observation and corresponds to a very extreme observation when *t* is large. We focus on the observations that are such that $r_{i}>1$. These provide a sub-sample of the standardized original sample. We define $v_{i}=z_{i}/r_{i}\in S_{\infty }^{d-1}$. These vectors are used for the estimation of Φ.

### 3.1. Projected Gamma Family

At the core of our PoT method is the development of a distribution on $S_{p}^{d-1}=\{s:s\in R_{+}^{d}{,∥s∥}_{p}=1\}$, where, for p>0, ${∥\cdot ∥}_{p}$ is the $L_{p}$-norm of a vector $x\in R^{d}$, defined as

$${∥x∥}_{p}={\left(\sum_{\ell =1}^{d}{\left|x_{\ell }\right|}^{p}\right)}^{\frac{1}{p}}.$$

The absolute and Euclidean norms are obtained for p=1 and p=2 respectively, and the $L_{\infty }$ norm can be obtained as a limit:

$${∥x∥}_{\infty }=\lim_{p\to \infty }∥x∥=\underset{\ell =1}{\overset{d}{⋁}}x_{\ell }.$$

**Figure 1:**
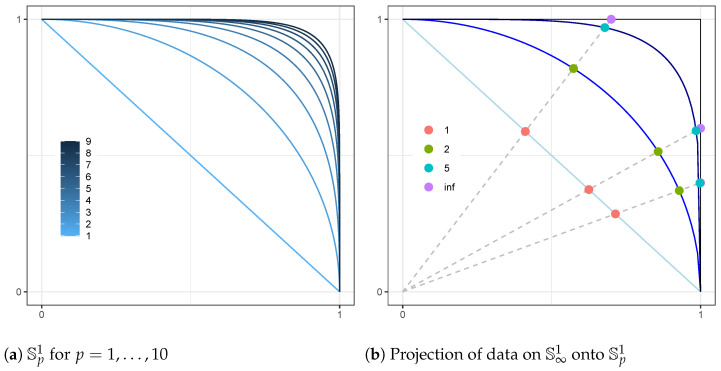
The positive orthant of the *p*-norm sphere for d=2.

To obtain a distribution on $S_{p}^{d-1}$, we start with a vector in $x\in R_{+}^{d}$ and normalize it to obtain $y=x/{∥x∥}_{p}\in S_{p}^{d-1}$. Figure 1a shows the progression of $S_{p}^{1}$ asymptotically towards $S_{\infty }^{1}$ as *p* increases; Figure 1b shows the relative positions of data points normalized to $S_{p}^{1}$ for selected *p*. A natural distribution to consider in $R_{+}^{d}$ is given by a product of independent univariate Gamma distributions. Let $X∼\prod_{\ell =1}^{d}\mathrm{Ga}\left(X_{\ell }|\alpha_{\ell },\beta_{\ell }\right)$. $\alpha_{\ell }$ and $\beta_{\ell }$ are the shape and scale parameters, respectively. For any finite p>0, letting $y_{d}={\left(1-\sum_{\ell =1}^{d-1}y_{\ell }^{p}\right)}^{1/p}$, the transformation

(3) $$T\left(x_{1},\ldots ,x_{d}\right)=\left({∥x∥}_{p},\frac{x_{1}}{{∥x∥}_{p}},\ldots ,\frac{x_{d-1}}{{∥x∥}_{p}}\right)=\left(r,y_{1},\ldots ,y_{d-1}\right)$$

is invertible with

(4) $$T^{-1}\left(r,y_{1},\ldots ,y_{d-1}\right)=\left(ry_{1},\ldots ,ry_{d-1},r{\left(1-\sum_{\ell =1}^{d-1}y_{\ell }^{p}\right)}^{\frac{1}{p}}\right).$$

The Jacobian of the transformation takes the form

(5) $$r^{d-1}\left[{\left(1-\sum_{\ell =1}^{d-1}y_{\ell }^{p}\right)}^{\frac{1}{p}}\right.+\left.\sum_{\ell =1}^{d-1}y_{\ell }^{p}{\left(1-\sum_{l=1}^{d-1}y_{\ell }^{p}\right)}^{\frac{1}{p}-1}\right].$$

The normalization provided by *T* maps a vector in $R_{+}^{d}$ onto $S_{p}^{d-1}$. With a slight abuse of terminology, we refer to it as a projection. Using Equations (3)–(5), we have the joint density

(6) $$f\left(r,y\right)=\prod_{\ell =1}^{d}\left[\frac{\beta_{\ell }^{\alpha_{\ell }}}{\Gamma (\alpha_{\ell })}{\left(ry_{\ell }\right)}^{\alpha_{\ell }-1}\exp\left\{-\beta_{\ell }ry_{\ell }\right\}\right]\times r^{d-1}\left[y_{d}+\sum_{\ell =1}^{d-1}y_{\ell }^{p}y_{d}^{1-p}\right].$$

Integrating out *r* yields the resulting *Projected Gamma* density

(7) $$\mathrm{PG}\left(y|\alpha ,\beta \right)=\prod_{\ell =1}^{d}\left[\frac{\beta_{\ell }^{\alpha_{\ell }}}{\Gamma (\alpha_{\ell })}y_{\ell }^{\alpha_{\ell }-1}\right]\times \left[y_{d}+\sum_{\ell =1}^{d-1}y_{\ell }^{p}y_{d}^{1-p}\right]\times \frac{\Gamma (\sum_{\ell =1}^{d}\alpha_{\ell })}{{\left(\sum_{\ell =1}^{d}\beta_{\ell }y_{\ell }\right)}^{\sum_{\ell =1}^{d}\alpha_{\ell }}},$$

defined for $y\in S_{p}^{d-1}$ and for any finite p>0. To avoid identifiability problems when estimating the shape and scale parameters, we set $\beta_{1}=1$. Ref. [20] obtain the density in Equation (7) for p=2 as a multivariate distribution for directional data using spherical coordinates. For $y\in S_{1}^{d-1}$ and $\beta_{\ell }=\beta$ for all *ℓ*, the density in Equation (7) corresponds to that of a Dirichlet distribution.

The projected gamma family is simple to specify and has very tractable computational properties. Thus, we use it as a building block for the angular measure Φ models. To build a flexible family of distributions in $S_{p}^{d-1}$, we consider mixtures of projected gamma densities defined as

(8) $$f\left(y\right)=\int_{\Theta }\mathrm{PG}\left(y|\theta \right)dG\left(\theta \right),$$

where θ=(α,β). Following a Bayesian non-parametric approach [21,22,23], we assume that *G* is drawn from a random measure. In particular, assuming a Dirichlet process prior for *G*, we have a hierarchical formulation of the mixture model that, for a vector of observations $y_{i}$, is given by

(9) $$y_{i}∼\mathrm{PG}\left(y_{i}|\theta_{i}\right)\;\theta_{i}∼G\;G∼\mathrm{DP}\left(\eta ,G_{0}\right)$$

where DP denotes a Dirichlet process, η is the precision parameter, and $G_{0}$ is the centering distribution.

Unfortunately, in the limit when p→∞, the normalizing transformation is not differentiable. Thus, a closed-form expression like Equation (7) for the projected gamma density on $S_{\infty }^{d-1}$ is not available. Instead, we observe that for a sufficiently large *p*, $S_{p}^{d-1}$ will approach $S_{\infty }^{d-1}$. With that in mind, our strategy consists of describing the angular distribution Φ using a sample-based approach with the following steps: (i) Apply the transformation in Equation (2) to the original data; (ii) Obtain the subsample of the standardized observations that satisfy R>1; (iii) Take a finite *p* and project the observations onto $S_{p}^{d-1}$; (iv) Fit the model in Equation (8) to the resulting data and obtain samples from the fitted model; (v) project the resulting samples onto $S_{\infty }^{d-1}$. For step (iv), we use a Bayesian approach that is implemented using a purposely developed Markov chain Monte Carlo sampler described in the next section.

### 3.2. Tail Probabilities for the PoT Model

A measure that is used to characterize the strength of the dependence in the tail for two random variables, $Z_{1}$ and $Z_{2}$, with marginal distributions $F_{1}$ and $F_{2}$ is given by [1]

$$\chi_{12}=\lim_{u↑1}\mathrm{Pr}\left[F_{1}\left(Z_{1}\right)>u|F_{2}\left(Z_{2}\right)>u\right].$$

$\chi_{12}$ provides information about the distribution of extremes for the variable $Z_{1}$ given that $Z_{2}$ is very large. When $\chi_{12}>0$, $Z_{1}$ and $Z_{2}$ are said to be asymptotically dependent; otherwise, they are asymptotically independent. The following result provides the asymptotic dependence coefficient between two components of Z for our proposed PoT model.

**Proposition** **1.** *Suppose that Z=RV with R∼Pa(1), $Pr\left[V_{\ell }>0\right]=1$ and $E\left[V_{\ell }\right]$ exists, for ℓ=1,…,d, then*

(10) $$\chi_{𝚥\ell }=E\left[\frac{V_{𝚥}}{E\left[V_{𝚥}\right]}\wedge \frac{V_{\ell }}{E\left[V_{\ell }\right]}\right]$$

**Proof.**  Denote as $F_{\ell }$ the marginal distribution of $Z_{\ell }$. To obtain $\chi_{𝚥\ell }$, we need $Pr(Z_{𝚥}>z_{𝚥},Z_{\ell }>z_{\ell })$, where $z_{\ell }=F_{\ell }^{-1}\left(u\right)=E\left[V_{\ell }\right]/\left(1-u\right),\;\ell =1,\ldots ,d$. Using the fact that $V_{\ell }>0,\forall \ell$ almost surely, we have that the former is equal to

$$\mathrm{Pr}\left[R>\frac{z_{𝚥}}{V_{𝚥}}\vee \frac{z_{\ell }}{V_{\ell }}\right]=E\left[1\wedge {\left(\frac{z_{𝚥}}{V_{𝚥}}\vee \frac{z_{\ell }}{V_{\ell }}\right)}^{-1}\right]=E\left[\frac{V_{𝚥}}{z_{𝚥}}\wedge \frac{V_{\ell }}{z_{\ell }}\right]=\left(1-u\right)E\left[\frac{V_{𝚥}}{E\left[V_{𝚥}\right]}\wedge \frac{V_{\ell }}{E\left[V_{\ell }\right]}\right]$$

where the second identity is justified by the fact that $V_{i}$ is bounded and $z_{i}\to \infty$. The proof is completed by noting that $\mathrm{Pr}\left[F_{i}\left(Z_{i}\right)>u\right]=1-u.$    □

Equation (10) implies that $\chi_{𝚥\ell }>0$, and so, no asymptotic independence is possible under our proposed model. For the analysis of extreme values, it is of interest to calculate the multivariate conditional survival function. The following result provides the relevant expression as a function of the angular measure.

**Proposition** **2.** *Assume the same conditions as Proposition 1. Let α⊂{1,…,d} be a collections of indexes. Then*

(11) $$\mathrm{Pr}\left[\underset{\ell \in \alpha }{⋂}Z_{\ell }>z_{\ell }|\underset{\ell \notin \alpha }{⋂}Z_{\ell }>z_{\ell }\right]=\frac{E\left[{⋀}_{k=1}^{d}1\wedge \frac{V_{k}}{z_{k}}\right]}{E\left[{⋀}_{k\notin \alpha }1\wedge \frac{V_{k}}{z_{k}}\right]}.$$

The proof uses a similar approach to the proof of Proposition 1.

Equations (10) and (11) provide relevant tools for inference on the tail behavior of the joint distribution of the observations. The expressions can be readily calculated within a sample-based inferential approach like the one considered in the following section.

#### Inference for the Projected Gamma Mixture Model

To perform inference for our proposed PoT model, we develop an iterative sample-based approach. We implement a Markov chain Monte Carlo method that, for a given iteration, groups observations into stochastically assigned clusters, where members of a cluster share distributional parameters [23,24]. Building out the methods of inference for Equation (9), let $n_{j}^{\left(-i\right)}$ be the number of observations in cluster *j*, not including observation *i*. Let $J^{\left(-i\right)}$ be the number of extant clusters, not including any singleton containing observation *i*. Under this model, the probability of cluster membership for a given observation is proportional to

$$\mathrm{Pr}\left[\delta_{i}=j|\ldots \right]\propto \left\{\begin{array}{c} n_{j}^{\left(-i\right)}\mathrm{PG}\left(y_{i}|\alpha_{j},\beta_{j}\right)\\ \eta \int \mathrm{PG}\left(y_{i}|\alpha_{j},\beta_{j}\right)dG_{0}\left(\alpha_{j},\beta_{j}\right), \end{array}\right.$$

where the top case is iterating over extant clusters $j=1,\ldots ,J^{\left(-i\right)}$, and the bottom case is for a *new* cluster. If $G_{0}$ is not a conjugate prior for the kernel density, the integral in the above formula may not be available in closed form. We sidestep this using Algorithm 8 from [25]: by Monte Carlo integration, we draw *m* candidate clusters, $\alpha_{j},\beta_{j}$ for $j=J^{\left(-i\right)}+1,\ldots ,J^{\left(-i\right)}+m$ from $G_{0}$. Then, we sample the cluster indicator $\gamma_{i}$ from extant or candidate clusters, where the probability of cluster membership is proportional to

(12) $$\mathrm{Pr}\left[\delta_{i}=j|\ldots \right]\propto \left\{\begin{array}{c} n_{j}^{\left(-i\right)}\mathrm{PG}\left(y_{i}|\alpha_{j},\beta_{j}\right)\\ \frac{\eta }{m}\mathrm{PG}\left(y_{i}|\alpha_{j},\beta_{j}\right). \end{array}\right.$$

Again, the top case is iterating over extant clusters, and now the bottom case is iterating over new *candidate* clusters. If a candidate cluster is selected, then $\gamma_{i}=J=J^{\left(-i\right)}+1$, and the associated cluster parameters are saved.

A key feature of the the projected Gamma distribution is its computational properties. We augment $\mathrm{PG}(y_{i}|\alpha_{i},\beta_{i})$ by introducing a latent radial component $r_{i}$, for each observation. Using Equation (6) we observe that the full conditional of $r_{i}$ is easy to sample from, as it is given as

(13) $$r_{i}|\alpha_{i},\beta_{i}∼G\left(r_{i}\;|\;\sum_{\ell =1}^{d}\alpha_{i\ell },\sum_{\ell =1}^{d}\beta_{\ell }y_{i\ell }\right).$$

Moreover, the full conditional for $\alpha_{j},\beta_{j}$ is then proportional to

(14) $$f\left(\alpha_{j},\beta_{j}|Y,r,\delta ,\ldots \right)\propto \prod_{i:\gamma_{i}=j}\prod_{\ell =1}^{d}G\left(r_{i}y_{i\ell }|\alpha_{j\ell },\beta_{j\ell }\right)\times dG_{0}\left(\alpha_{j},\beta_{j}\right).$$

Note that the ordering of the products can be reversed in Equation (14), indicating that with appropriate choice of centering distribution, the full conditionals for $\alpha_{j},\beta_{j}$ can become separable by dimension, and thus inference on $\alpha_{j\ell },\beta_{j\ell }$ can be done in parallel for all *j*, *ℓ*. We first consider a centering distribution given by a product of independent Gammas:

(15) $$G_{0}\left(\alpha_{j},\beta_{j}|\xi ,\tau ,\zeta ,\sigma \right)=\prod_{\ell =1}^{d}G\left(\alpha_{j\ell }|\xi_{\ell },\tau_{\ell }\right)\times \prod_{\ell =2}^{d}G\left(\beta_{j\ell }|\zeta_{\ell },\sigma_{\ell }\right).$$

This model is completed with independent Gamma priors on $\xi_{\ell }$, $\tau_{\ell }$, $\zeta_{\ell }$, and $\sigma_{\ell }$. We also assume a Gamma prior on η, which is updated via the procedure outlined in [26]. We refer to this model as the *projected gamma-gamma* (PG-G) model. An advantage of the PG-G model is that, thanks to conjugacy, the rate parameters $\beta_{j\ell }$ can easily be integrated out for inference on $\alpha_{j}$. Then, the full conditional for $\alpha_{j\ell }$ takes the form

(16) $$\pi \left(\alpha_{j\ell }|r,Y,\gamma ,\xi_{\ell },\tau_{\ell },\zeta_{\ell },\sigma_{\ell }\right)\propto \frac{{\left(\prod_{i:\gamma_{i}=j}r_{i}y_{i\ell }\right)}^{\alpha_{j\ell }-1}\alpha_{j\ell }^{\xi_{\ell }-1}e^{-\tau_{\ell }\alpha_{j\ell }}}{\Gamma^{n_{j}}\left(\alpha_{j\ell }\right)}\times \frac{\Gamma \left(n_{j}\alpha_{j\ell }+\zeta_{\ell }\right)}{{\left(\sum_{i:\gamma_{i}=j}r_{i}y_{i\ell }+\sigma_{\ell }\right)}^{n_{j}\alpha_{j\ell }+\zeta_{\ell }}}$$

for ℓ=2,…,d. For ℓ=1, as $\beta_{1}:=1$, the full conditional takes the simpler form

(17) $$\pi \left(\alpha_{j1}|r,Y,\gamma ,\xi_{1},\tau_{1}\right)\propto \frac{{\left(\prod_{i:\gamma_{i}=j}r_{i}y_{i1}\right)}^{\alpha_{j1}-1}\alpha_{j1}^{\xi_{1}-1}e^{-\tau_{1}\alpha_{j1}}}{\Gamma^{n_{j}}\left(\alpha_{j1}\right)}.$$

Samples of $\alpha_{j\ell }$ can thus be obtained using a Metropolis step. In our analysis, we first transform $\alpha_{j\ell }$ to the log scale and use a normal proposal density. The full conditional for β is

(18) $$\beta_{j\ell }|r,Y,\alpha ,\zeta_{\ell },\sigma_{\ell }∼G\left(\beta_{j\ell }\;|\;n_{j}\alpha_{j\ell }+\zeta_{\ell },\sum_{i:\gamma_{i}=j}r_{i}y_{i\ell }+\sigma_{\ell }\right),$$

for ℓ=2,…,d. Updating $\beta_{j\ell }$ is done via a Gibbs step. The hyper-parameters $\xi_{\ell },\tau_{\ell },\zeta_{\ell },\sigma_{\ell }$ follow similar gamma-gamma update relationships. We also explore a restricted form of this model, where $\beta_{\ell }=1$ for all *ℓ*. Under this model, we use the full conditional in Equation (17) for all *ℓ* and omit inference on ζ,σ. We refer to this model as the *projected restricted gamma-gamma* (PRG-G) model.

The second form of centering distribution we explore is a multivariate log-normal distribution on the shape parameters $\alpha_{j}$ with independent gamma $\beta_{j\ell }$ rate parameters.

(19) $$G_{0}\left(\alpha_{j},\beta_{j}|\mu ,\Sigma ,\zeta ,\sigma \right)=\mathrm{LN}\left(\alpha_{j}|\mu ,\Sigma \right)\times \prod_{\ell =2}^{d}G\left(\beta_{j\ell }|\zeta_{\ell },\sigma_{\ell }\right).$$

This model is completed with a normal prior on μ, an inverse Wishart prior on Σ, and Gamma priors on $\zeta_{\ell }$, $\sigma_{\ell }$, and η. This model is denoted as the *projected gamma-log-normal* (PG-LN) model. We also explore a restricted Gamma form of this model as above, where $\beta_{\ell }=1$ for all *ℓ*. This is denoted as the *projected restricted gamma-log-normal* (PRG-LN) model. Updates for α can be accomplished using a joint Metropolis step, where $\beta_{j\ell }$ for ℓ=2,…,d have been integrated out of the log-density. That is,

$$\begin{array}{cc} \pi (\alpha_{j}|Y,r,\delta ,\mu ,\Sigma ,\zeta ,\sigma ) & \propto \exp\left\{-\frac{1}{2}{\left(\log\alpha_{j}-\mu \right)}^{T}\Sigma^{-1}\left(\log\alpha_{j}-\mu \right)\right\}\times \frac{1}{\prod_{\ell =1}^{d}\alpha_{j\ell }}\\ & \;\times \frac{{\left(\prod_{i:\gamma_{i}=j}r_{i}y_{i1}\right)}^{\alpha_{j1}-1}}{\prod_{\ell =1}^{d}\Gamma^{n_{j}}\left(\alpha_{j\ell }\right)}\times \prod_{\ell =2}^{d}\frac{\Gamma \left(n_{j}\alpha_{j\ell }+\zeta_{\ell }\right)}{{\left(\sum_{i:\gamma_{i}=j}r_{i}y_{i\ell }+\sigma_{\ell }\right)}^{n_{j}\alpha_{j\ell }+\zeta_{\ell }}} \end{array}$$

The inferential forms for $\beta_{j\ell }$ and its priors are the same as for PG-G. The normal prior for μ is conjugate for the log-normal $\alpha_{j}$ and can be sampled via a Gibbs step. Finally, the inverse Wishart prior for Σ is again conjugate to the log-normal $\alpha_{j}$, implying that it can also be sampled via a Gibbs step.

To effectively explore the sample space with a joint Metropolis step, as well as to speed convergence, we implement a parallel tempering algorithm [27] for the log-normal models. This technique runs parallel MCMC chains at ascending temperatures. That is, for chain *s*, the posterior density is exponentiated by the reciprocal of temperature $t_{s}$. For the *cold* chain, $t_{1}:=1$. Let $E_{s}$ be the log-posterior density under the current parameter state for chain *s*. Then states between chains r,s are exchanged via a Metropolis step with probability

$$\alpha_{rs}=\min\left[1,\exp\left\{\left(t_{r}^{-1}-t_{s}^{-1}\right)\left(E_{r}-E_{s}\right)\right\}\right].$$

Higher temperatures serve to *flatten* the posterior distribution, meaning hotter chains have a higher probability of making a given transition or will make larger transitions. As such, they will more quickly explore the parameter space and share information gained through state exchange.

## 4. Scoring Criteria for Distributions on the Infinity-Norm Sphere

In order to assess and compare the estimation of a distribution on $S_{\infty }^{d-1}$, we consider the theory of proper scoring rules developed in [28]. As mentioned in Section 3.1, our approach does not provide a density on $S_{\infty }^{d-1}$, restricting our ability to construct model selection criteria to sample-based approaches. To this end, we employ the *energy score* criterion introduced therein.

The energy score criterion, defined for a general probability distribution *P* with a finite expectation, is developed as

(20) $$S_{\mathrm{ES}}\left(P,x_{i}\right)=E_{p}\left[g\left(X_{i},x_{i}\right)\right]-\frac{1}{2}E_{p}\left[g\left(X_{i},X_{i}^{\prime }\right)\right],$$

where *g* is a kernel function. The score defined in Equation (20) can be evaluated using samples from *P* with the help of the law of large numbers. Moreover, Theorem 4 in [28] states that if g(·,·) is a negative definite kernel, then S(P,x) is a *proper* scoring rule. Recall that a real valued function *g* is a negative definite kernel if it is symmetric in its arguments, and $\sum_{i=1}^{n}\sum_{j=1}^{n}a_{i}a_{j}g\left(x_{i},x_{j}\right)\leq 0$ for all positive integers *n* and any collection $a_{1},\ldots ,a_{n}\in R$ such that $\sum_{i=1}^{n}a_{i}=0$.

In a Euclidean space, these conditions are satisfied by the Euclidean distance [29]. However, for observations on different faces of $S_{\infty }^{d-1}$, the Euclidean distance will under-estimate the geodesic distance, the actual distance required to travel between the two points. Let

$$C_{\ell }^{d-1}=\left\{x:x\in S_{\infty }^{d-1},x_{\ell }=1\right\}$$

comprise the *ℓ*th *face*. For points on the same face, the Euclidean distance corresponds to the length of the shortest possible path in $S_{\infty }^{d-1}$. For points on different faces, the Euclidean distance is a lower bound for that length.

For a finite *p*, the shortest connecting path between two points in $S_{p}^{d-1}$ is the minimum geodesic; its length satisfying the definition of a distance. Thus its length can be used as a negative definite kernel for the purpose of defining an energy score. Unfortunately, as p→∞, the resulting surface $S_{\infty }^{d-1}$ is not differentiable, implying that routines to calculate geodesics are not readily available. However, as $S_{\infty }^{d-1}$ is a portion of a *d*-cube, we can borrow a result from geometry [30] stating that the length of the shortest path between two points on a geometric figure corresponds to the length of a straight line drawn between the points on an appropriate unfolding, rotation, or *net* of the figure from a *d*-dimensional to a d−1-dimensional space. The optimal net will have the shortest straight line between the points, as long as that line is fully contained within such a net. As $S_{\infty }^{d-1}$ has *d* faces—each face pairwise adjacent, there are d! possible nets. However, we are only interested in nets that begin and end on the source and destination faces, respectively, reducing the number of nets under consideration to ∑k=0d−2d−2k. This is still computationally burdensome for a large number of dimensions. However, we can efficiently establish an upper bound on the geodesic length. We use this upper bound on geodesic distance as the kernel function for the energy score.

To calculate the energy score, we define the kernel

$$g\left(a,b\right)=\min_{c\in C_{𝚥}^{d-1}\cap C_{\ell }^{d-1}}\left\{{∥c-a∥}_{2}+{∥b-c∥}_{2}\right\}.$$

where $a\in C_{\ell }^{d-1}$, and $b\in C_{𝚥}^{d-1}$ for ℓ,𝚥∈{1,…,d}. Evaluating *g* as described requires the solution of a (d−2)-dimensional optimization problem. The following proposition provides a straightforward approach.

**Proposition** **3.** *Let $a\in C_{\ell }^{d-1}$, and $b\in C_{𝚥}^{d-1}$, for ℓ,𝚥∈{1,…,d}. For ℓ≠𝚥, the transformation $P_{𝚥\ell }\left(\cdot \right)$ required to rotate the 𝚥th face along the ℓth axis produces the vector $b^{\prime }$, where*

(21) $$b_{i}^{\prime }=P_{𝚥\ell }\left(b\right)=\left\{\begin{array}{cc} b_{i} & \mathrm{for}\;i\neq 𝚥,\ell \\ 1 & \mathrm{for}\;i=\ell \\ 2-b_{\ell } & \mathrm{for}\;i=𝚥 \end{array}\right..$$

*Then $g\left(a,b\right)={∥a-b^{\prime }∥}_{2}$.*

**Proof.**  Notice that for $c\in C_{𝚥}^{d-1}\cap C_{\ell }^{d-1}$, ${∥b-c∥}_{2}={∥b^{\prime }-c∥}_{2}$. We then have that

$$\begin{array}{cc} g(a,b) & =\min_{c\in C_{𝚥}^{d-1}\cap C_{\ell }^{d-1}}\left\{{∥c-a∥}_{2}+{∥b-c∥}_{2}\right\}\\ \; & =\min_{c\in C_{𝚥}^{d-1}\cap C_{\ell }^{d-1}}\left\{{∥c-a∥}_{2}+{∥b^{\prime }-c∥}_{2}\right\}\\ \; & ={∥a-b^{\prime }∥}_{2}. \end{array}$$

The last equality is due to the fact that a and $b^{\prime }$ belong to the same hyperplane.    □

Using the rotation in Proposition 3, we obtain the following result.

**Proposition** **4.** *g is a negative definite kernel.*

**Proof.**  For a given *n*, consider an arbitrary set of points $a_{1},\ldots ,a_{n}\in S_{\infty }^{d-1}$, and $\alpha_{1},\ldots ,\alpha_{n}\in R$, such that $\sum_{i=1}^{n}\alpha_{i}=0$. Then

$$\sum_{i,𝚥}\alpha_{i}\alpha_{𝚥}g\left(a_{i},a_{𝚥}\right)=\sum_{i,𝚥}\alpha_{i}\alpha_{𝚥}{∥a_{i}-a_{𝚥}^{\prime }∥}_{2}\leq 0,$$

where $a_{𝚥}^{\prime }$ is defined as in Proposition 3. The last equality holds as $∥x-x^{\prime }{∥}_{2},x,x^{\prime }\in R^{d}$ is negative definite [28]   □

Proposition 3 provides a computationally efficient way to evaluate the proper scoring rule $S_{\mathrm{ES}}$ defined on $S_{\infty }^{d-1}$ for each observation. For the purpose of model assessment and comparison, we report the average $S_{\mathrm{ES}}$ taken across all observed data and notice that the smaller the score, the better.

## 5. Data Illustrations

We apply the aforementioned models to simulated angular data. We then consider the analysis of atmospheric data. To tackle the difficult problem of assessing the convergence an MCMC chain for a large-dimensional model, we monitor the log-posterior density. In all the examples considered, MCMC samples produced stable traces of the log-posterior in less than 40,000 iterations. We use that as a burn-in and thereafter sample 10,000 additional iterations. We then thin the chain by retaining one every ten samples, to obtain 1000 total samples. These are used to generate samples from the posterior predictive densities. We used two different strategies to implement the MCMC samplers. For the models whose DP prior is centered around a log-normal distribution, we used parallel tempering. This serves to overcome the very slow mixing that we observed in these cases. The temperature ladder was set as $t_{s}=1.3^{s}$ for s∈{0,1,…,5}. This was set empirically in order to produce acceptable swap probabilities both for the simulated data and real data. Parallel tempering produces chains with good mixing properties but has a computational cost that grows linearly with the number of temperatures. Thus, for the gamma-centered models, we used a single chain. We leverage the fast speed of each iteration to obtain a large number of samples, which are appropriately thinned to deal with a mild autocorrelation. In summary, the strategy for log-normal centered models is based on a costly sampler with good mixing properties. The strategy for the gamma-centered models is based on a cheap sampler that can be run for a large number of iterations.

Our hyperprior parameters are set as follows: For the gamma-centered models (PG-G, PRG-G), the shape parameter for the centering distribution $\xi_{\ell }∼G\left(1,1\right)$, and rate parameter $\tau_{\ell }∼G\left(2,2\right)$. For the log-normal centered models (PG-LN, PRG-LN), the centering distribution’s log-mean $\mu ∼N_{d}\left(0,I_{d}\right)$, and covariance matrix $\Sigma ∼\mathrm{IW}\left(d+10,\left(d+10\right)I_{d}\right)$. These values are intended such that draws from the prior for Σ will weakly tend towards the identity matrix. For models learning rate parameters $\beta_{j\ell }$ (PG-G, PG-LN), the centering distribution’s shape parameter $\zeta ∼G\left(1,1\right)$ and rate parameter $\sigma ∼G\left(2,2\right)$. The choice of the G(2,2) for rate parameters places little mass near 0 in order to draw estimates for the value away from 0 for numerical stability.

### 5.1. Simulation Study

The challenging problem in multivariate EVT is to capture the dependence structure of the limiting distribution. To this end, we focus our simulation study specifically on the angular component. To evaluate our proposed approach for angular measure estimation, we consider simulated datasets on $S_{\infty }^{d-1}$ for values of *d* ranging from 2 to 32. We generated each dataset as a mixture of multivariate log-normal distributions projected onto $S_{\infty }^{d-1}$. The generation procedure is detailed in Algorithm 1. We produced ten replicates of each configuration. We consider two gamma-centered and two log-normal-centered DP mixture models, with and without restrictions in each case. To perform a comparative analysis, we fitted the pairwise betas model proposed in [31]. We chose this model for comparison, as it similarly works to capture a complex dependence structure on an $S_{p}^{d-1}$ sphere, albeit with p=1, and is implemented in the readily available package BMAmevt in R [32], which can provide samples from the posterior predictive distribution. These samples are needed for the calculation of the energy scores that are at the basis of our comparison. In addition, BMAmevt can be fitted to moderately large multivariate observations. For the DP mixture models, the data are projected onto $S_{10}^{d-1}$. For the other two models, they are projected onto $S_{1}^{d-1}$. We sampled each model for 50,000 iterations, dropping the first 40,000 as burn-in and thinning to keep every 10th iteration after. These settings were intended to provide a consistent sampling strategy that would work with every model, even if inefficient for some.
**Algorithm 1** Simulated Angular Dataset Generation Routine. $\mu_{j}$, $\Sigma_{j}$ are the parameters of the mixture component distribution; π is the probability vector assigning weight mixture components; $\delta_{i}$ is the mixture component identifier for each simulated observation.**for** $n_{\mathrm{iter}}$ in [1,…,10] **do**    **for** $n_{\mathrm{mix}}$ in [1,2,4,8] **do**        **for** *j* in $1,\ldots ,n_{\mathrm{mix}}$ **do**           Generate $\mu_{j}∼N_{32}\left(0,I\right)$           Generate $\Sigma_{j}∼{\mathrm{IW}}_{32}\left(70,70I\right)$        **end for**        Generate $\pi ∼\mathrm{Dirichlet}(10_{n_{\mathrm{mix}}})$        **for** *i* in 1,…,1000 **do**           Generate $\delta_{i}∼\mathrm{Categorical}\left(\pi \right)$           Generate $X_{i}∼\mathrm{LN}\left(\mu_{\left[\delta_{i}\right]},\Sigma_{\left[\delta_{i}\right]}\right)$        **end for**        **for** $n_{\mathrm{col}}$ in [2,4,8,16,24,32] **do**           Project columns 1 to $n_{\mathrm{col}}$ of X onto $S_{\infty }^{n_{\mathrm{col}}-1}$ and save.        **end for**    **end for****end for**

**Figure 2:**
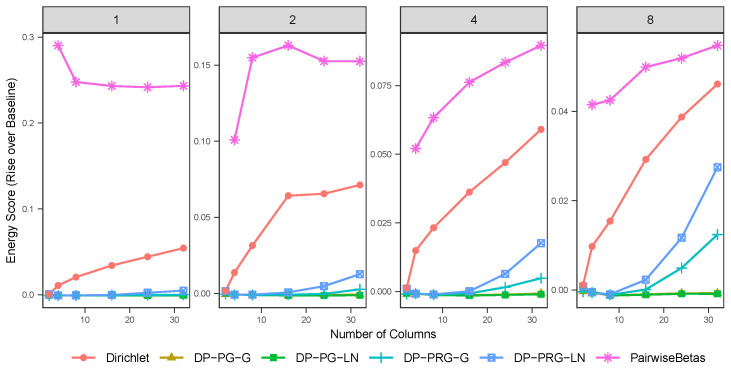
Average energy score rise over baseline (on $S_{\infty }^{d-1}$) for various models fitted to simulated data, with ascending count of mixture components (indicated by plot heading) and number of dimensions (indicated by horizontal axis). Note that pairwise betas is a moment-restricted model.

Figure 2 shows the average rise over baseline in energy score, as calculated on $S_{\infty }^{d-1}$ using the kernel metric described in Proposition 3, for models trained on simulated data. After training a model, a posterior predictive dataset is generated, and the energy score is calculated as a Monte Carlo approximation of Equation (20). In our analysis, after burn-in and thinning, we had 1000 replicates from the posterior distribution and generated 10 posterior predictive replicates per iteration. The *baseline* value is the energy score of a new dataset from the same generating distribution as the training dataset evaluated against the training dataset. For the simulated data, we observe that the projected gamma models dominate the other two options considered, regardless of the choice of centering distribution. The projected restricted gamma models with a multivariate log-normal centering distribution appear to be dominated by the models based on the alternative centering distributions. Moreover, the performance deteriorates with the increase in dimensionality. Additionally, models centered around the log-normal distribution incur the computational cost of multivariate normal evaluation and parallel tempering, taking approximately six times longer to sample relative to the gamma models. We also note that the computational cost of the pairwise betas model grows combinatorially, with a sample space of dimension d2+1. By comparison, the sample space for PG-G and PRG-G are 2(J+1)d and (J+1)d, respectively, where *J* is the number of extant clusters, with much of that inference able to be done in parallel. In our testing, for low-dimensional problems, BMAmevt was substantially faster than any of our proposed DP mixture models. However, for examples with high numbers of dimensions, the computational time for BMAmevt was greater than that for PG-G. We compare computing times in our data analysis in Table 1b.

### 5.2. Integrated Vapor Transport

The *integrated vapor transport* (IVT) is a two-component vector that tracks the flow of the total water volume in a column of air over a given area [33]. IVT is increasingly used in the study of atmospheric rivers because of its direct relationship with orographically induced precipitation [34]. Atmospheric rivers (AR) are elongated areas of high local concentration of water vapor in the atmosphere that transport water from the tropics around the world. AR can cause extreme precipitation, something that is usually associated with very large values of the IVT magnitude over a whole geographical area. In spite of this, AR are fundamental for the water supply of areas like California. Thus, the importance of understanding the extreme behavior of IVT includes extreme tail dependence. We consider datasets that correspond to IVT estimated at two different spatial resolutions. The coarse-resolution dataset is obtained from the European Centre for Medium-Range Weather Forecasts (ECMWF) Interim reanalysis (ERA-Interim) [35,36]. The high-resolution dataset corresponds to the latest ECMWF observational product, ERA5 [37].

**Figure 3:**
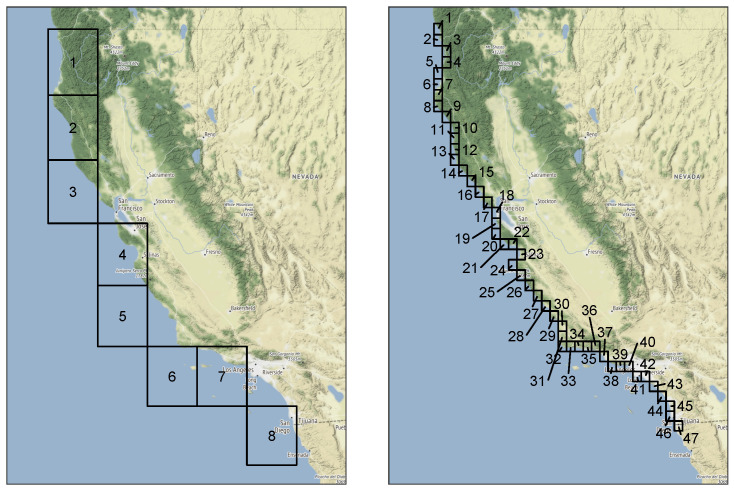
Grid cell locations for ERA-Interim (**left**) and ERA5 (**right**).

Our data correspond to daily average values for the IVT magnitude along the coast of California. The ERA-Interim data used cover the time period 1979 through 2014 (37 years), omitting leap days, and eight grid cells that correspond to the coast of California. The ERA5 data cover the time period 1979 through 2019 (42 years) with the same restriction and 47 grid cells for the coast of California. This gives us the opportunity to illustrate the performance of our method in multivariate settings of very different dimensions. Figure 3 provides a visual representation of the area these grid cells cover.

Fitting our models to the IVT data requires some pre-processing. First, we subset the data to the rainy season, which in California runs roughly from November to March. Following the approach described in Section 3, we estimate the shape and scale parameters of a univariate GP in each dimension, using maximum likelihood. We set the threshold in each dimension *ℓ* as $b_{t,\ell }={\hat{F}}_{\ell }^{-1}\left(1-t^{-1}\right)$, where $\hat{F}$ is the empirical CDF and t=20, which corresponds to the 95 percentile. We then use the transformation in Equation (2) to standardize the observations. Dividing each standardized observation by its $L_{\infty }$ norm, we obtain a projection onto $S_{\infty }^{d-1}$. As the data correspond to a daily time series, the observations are temporally correlated. For each group of consecutive standardized vectors $z_{i}$ such that ${∥z_{i}∥}_{\infty }>1$, we retain only the vector with the largest $L_{\infty }$ norm. The complete procedure is outlined in Algorithm 2.

**Figure 4:**
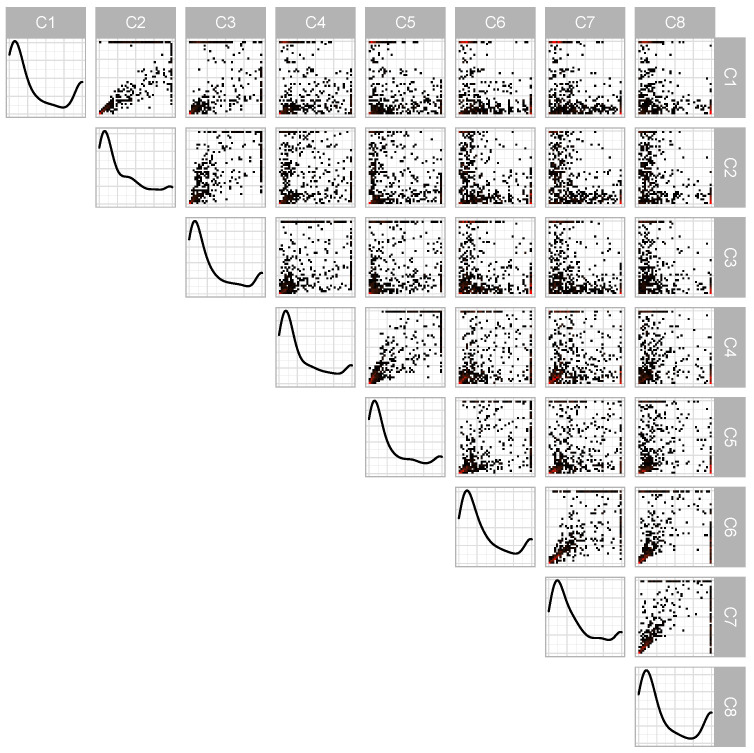
Pairwise plots from ERA-Interim data after transformation and projection to $S_{\infty }^{7}$. Down the diagonal are marginal kernel densities with two-dimensional histograms on the off-diagonal. In those plots, red indicates a higher density. All data are between 0 and 1.

After subsetting the ERA-Interim data to the rainy season, we have 5587 observations. After the processing and declustering described in Algorithm 2, this number reduces to 511 observations. A pairwise plot of the transformed data after processing and declustering is presented in Figure 4. From this, we note that the marginal densities display strong similarities, with a large spike near 0 and a small spike near 1. A value of 1 in a particular axis indicates that the standardized threshold exceedance was the largest in that dimension. The off-diagonal plots correspond to pairwise density plots. We observe that some site pairs, such as (1,2), (7,8), and especially (4,5), have the bulk of their data concentrated in a small arc along the $45^{\circ }$; in other site combinations, such as (3,6), (2,7), or (1,8), the data is split, favoring one side or the other of the $45^{\circ }$ line. For the ERA5 data, after subsetting, we have 6342 observations, which reduces to 532 observations after processing and declustering. We fit the PG-G, PRG-G, PG-LN, and PRG-LN models to both datasets.
**Algorithm 2** Data preprocessing to isolate and transform data exhibiting extreme behavior. $r_{i}$ represents the radial component, and $v_{i}$ the angular component. The declustering portion is relevant for data correlated in time.**for** ℓ=1,…,d **do**    Set $b_{t,\ell }={\hat{F}}_{\ell }^{-1}\left(1-\frac{1}{t}\right)$.    With $x_{\ell }>b_{t,\ell }$, fit $\sigma_{\ell }$, $\xi_{\ell }$ via MLE according to generalized Pareto likelihood.**end for****for** i=1,…,n **do**    Define $z_{i,\ell }={\left(1+\xi_{\ell }\frac{x_{i,\ell }-b_{t,\ell }}{\sigma_{\ell }}\right)}_{+}^{1/\xi_{\ell }}$;      then $r_{i}={∥z_{i}∥}_{\infty }$, $\;\;v_{i}=\frac{z_{i}}{{∥z_{i}∥}_{\infty }}$**end for**Subset r,v such that $r_{i}\geq 1$**if** declustering **then**    **for** i=1,…,n **do**        If $r_{i}\geq 1$ and $r_{i-1}\geq 1$, drop the lesser (and associated $v_{i}$) from dataset.    **end for****end if**

Table 1a shows the values of the estimated energy scores for the different models considered. We observe that, contrary to the results in the simulation study in Figure 2, the preferred model is the projected restricted gamma models, though for the lower-dimensional ERA-Interim data, all models perform comparably. Table 1b shows the computing times needed to fit the different models to the two datasets. We see the effect of dimensionality on the various models; for gamma-centered models, it grows linearly; for the log-normal centered model, it will grow superlinearly as matrix inversion becomes the most costly operation. For BMAmevt, its parameter space grows combinatorially with the number of dimensions, and thus so does computational complexity and sampling time.

**Figure 5:**
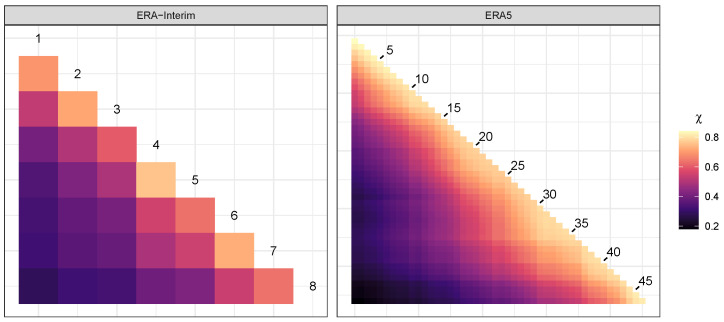
Pairwise extremal dependence coefficients for IVT data using the PRG-G model.

We consider an exploration of the pairwise extremal dependence using Monte Carlo estimates of the coefficients in Equation (10). For this, we use samples obtained from the PRG-G model. Figure 5 provides a graphical analysis of the results. The coefficients achieve values between 0.286 and 0.759 for the ERA-Interim data and between 0.181 and 0.840 for the ERA5 data. The greater range in dependence scores observed in the ERA5 data versus ERA-Interim speaks to the greater granularity of the ERA5 data, indicating that distance between locations is a strong contributor to the strength of the pairwise asymptotic dependence. The highest coefficients are 0.759 for locations 4 and 5 in the ERA-Interim data and 0.840 for locations 1 and 2 in the ERA5 data. Clearly, pairwise asymptotic dependence coefficients tell a limited story, as a particular dependence may include more than two locations. We can, however, glean some information from the patterns that emerge in two dimensions. For the ERA-Interim data, we observe a possible cluster between cells 5–8, indicating a strong dependence among these cells. Analogously, for the ERA5 data, we observe three possible groups of locations.

**Figure 6:**
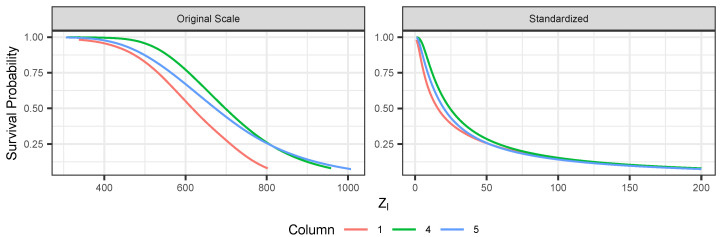
Conditional survival curves for selected locations using ERA-Interim and PRG-G model conditioning on all other dimensions at greater than 90th percentile (fitted). The left panel uses original units. Right panel uses standardized units.

**Figure 7:**
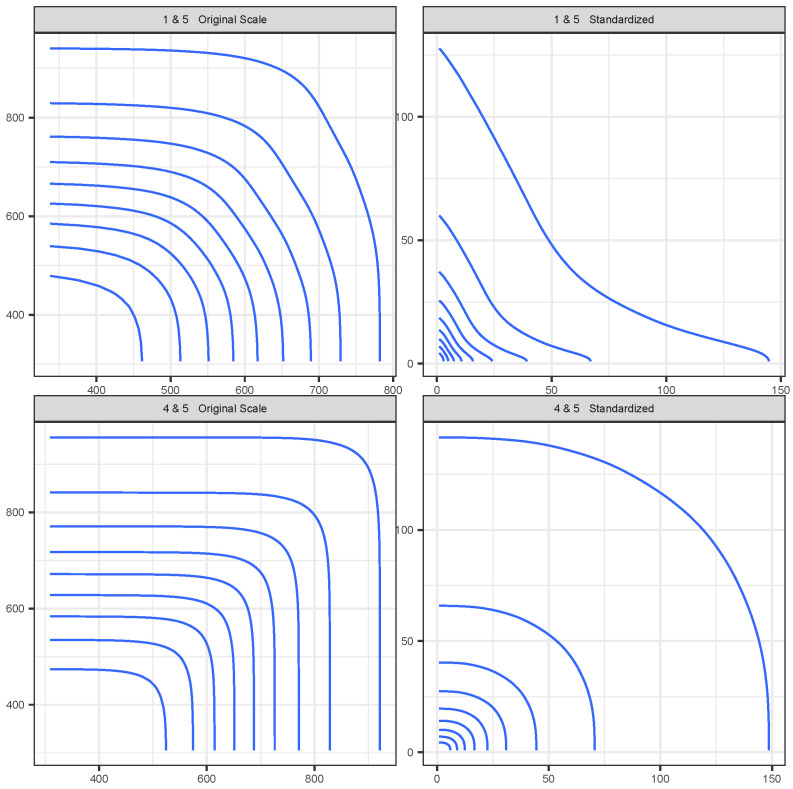
Pairwise conditional survival curves for selected locations, using ERA-Interim and PRG-G models, conditioning on all other dimensions at greater than the 90th percentile (fitted).

Figure 6 shows, for the ERA-Interim data under the PRG-G model, the conditional survival curve defined in Equation (11) for one dimension conditioned on all other dimensions being greater than their (fitted) 90th percentile. Figure 7 presents the bi-variate conditional survival function, conditioning on all other dimensions. These results illustrate quantitatively how extremal dependence affects the shape of the conditional survival curves. The two top panels represent the joint survival function between grid locations 4 and 5, which are shown in Figure 5 to exhibit strong extremal dependence. We observe that the joint survival surface is strongly convex. The bottom panels represent the joint survival surface between grid locations 1 and 5, which exhibited low extremal dependence. In this case, the shape of the contours tends to be concave, quite different from the shapes observed in the top panels.

Using our proposed scoring criteria, we explored the effect of the choice of *p* on the final results. Using the simulated data, generated from a mixture of projected gammas, we were unable to observe sizeable differences in the scores for *p* ranging between 1 and 15. However, for the IVT data, we observed a drop in the energy score associated with higher *p*, with a diminishing effect as *p* increased. We observed no significant differences in the performance of the model that uses p=10, which corresponds to the analysis presented, relative to the one that uses p=15.

## 6. Conclusions

In this paper, we have built upon the definition of the multivariate Pareto described in [11] to establish a useful representation of its dependence structure through the distribution of its angular component, which is supported on the positive orthant of the unit hypersphere under the $L_{\infty }$ norm, $S_{\infty }^{d-1}$. Due to the inherent difficulty of obtaining the likelihood of distributions with support on $S_{\infty }^{d-1}$, our method transforms the data to $S_{p}^{d-1}$, fits then using mixtures of products of independent gammas, then transforms the predictions back to $S_{\infty }^{d-1}$. As $S_{p}^{d-1}$ converges to $S_{\infty }^{d-1}$ as p→∞, we expect the proposed resampling to be efficient for large enough *p*. In fact, our exploration of the simulated and real data indicates that the procedure is robust to the choice of moderately large values of *p*. Our method includes two inferential steps. The first consists of the estimation of the marginal Pareto distributions; the second consists of the estimation of the angular density. Parameter uncertainty incurred in the former is not propagated to the latter. Conceptually, an integrated approach that accounts for all the estimation uncertainty is conceivable. Unfortunately, this leads to posterior distributions with complex data-dependent restrictions that are very difficult to explore, especially in large dimensional settings. In fact, our attempts to fit a simple parametric model for the marginals and the angular measures jointly in several dimensions were not successful.

In this paper, we have focused on a particular representation of the multivariate Pareto distribution for PoT inference on extreme values. To this end, our model provides a computationally efficient and flexible approach. An interesting extension of the proposed model is to consider regressions of extreme value responses, due to extreme value inputs following the ideas in [38]. This will produce PoT-based Bayesian non-parametric extreme value regression models. More generally, models that allow for covariate-dependent extremal dependence [39] could be considered. In addition, we notice that our approach is based on flexibly modeling angular distributions for any *p*-norm. As such, it can be applied to other problems focused on high-dimensional directional statistics constrained to a cone of directions.

Developing an angular measure specifically in $S_{\infty }^{d-1}$ provides two benefits over $S_{p}^{d-1}$. First, the transformation to $S_{\infty }^{d-1}$ is unique. Recall that Equation (4) gives $y_{d}$ as a function of $y_{1},\ldots ,y_{d-1}$. An analogous expression can be obtained for any $y_{\ell }$. This indicates that there are *d* equivalent transformations, each yielding a different Jacobian and, for p>1, potentially resulting in a different density. Second, the evaluation of geodesic distances on $S_{p}^{d-1}$ is not straightforward. However, we have demonstrated a computationally efficient upper bound on geodesic distance on $S_{\infty }^{d-1}$. Accepting these foibles, it would be interesting to explore the distribution of $S_{p}^{d-1}$,

The computations in this paper were performed on a desktop computer with an AMD Ryzen 5000 series processor. The program is largely single-threaded, so computation time is not dependent on the available core count. In each case, we run the MCMC chain for 50,000 iterations, with a burn-in of 40,000 samples. Fitting the PG-G model on the ERA5 dataset took approximately 15 min. Work is in progress to optimize the code and explore parallelization where possible. We are also exploring alternative computational approaches that will make it feasible to tackle very high dimensional problems, such as variational Bayes. In fact, to elaborate on the study of IVT, there is a need to consider several hundreds, if not thousands, of grid cells over the Pacific Ocean in order to obtain a good description of atmospheric events responsible for large storm activity over California.

## Figures and Tables

**Table 1:** Model fit assessment and computation time on ERA-Interim and ERA5 data. (a) Energy score criterion from fitted models against the IVT data. Lower is better. (b) Time to sample (in minutes) 50,000 iterations for various models.

(a)					
**Source**	**Pairwise Betas**	**PG-G**	**PG-LN**	**PRG-G**	**PRG-LN**
ERA-Interim	0.8620	0.8003	0.7986	**0.7966**	0.7970
ERA5	2.0311	1.6404	1.5576	**1.4349**	1.5051
(**b**)					
**Source**	**Pairwise Betas**	**PG-G**	**PG-LN**	**PRG-G**	**PRG-LN**
ERA-Interim	1.5	16.3	66.5	14.8	52.9
ERA5	53.1	19.4	153.4	24.6	121.4

## Data Availability

The raw data supporting the conclusions of this article will be made available by the authors on request.
